# Method for Aluminum Oxide Thin Films Prepared through Low Temperature Atomic Layer Deposition for Encapsulating Organic Electroluminescent Devices

**DOI:** 10.3390/ma8020600

**Published:** 2015-02-10

**Authors:** Hui-Ying Li, Yun-Fei Liu, Yu Duan, Yong-Qiang Yang, Yi-Nan Lu

**Affiliations:** Computer Science and Technology Department, Jilin University, Changchun 130012, Jilin, China; E-Mails: liyray@gmail.com (Y.-F.L.); duanyu@jlu.edu.cn (Y.D.); yangyq12@mails.jlu.edu.cn (Y.-Q.Y.)

**Keywords:** atomic layer deposition, lower temperature, uniform thin film

## Abstract

Preparation of dense alumina (Al_2_O_3_) thin film through atomic layer deposition (ALD) provides a pathway to achieve the encapsulation of organic light emitting devices (OLED). Unlike traditional ALD which is usually executed at higher reaction n temperatures that may affect the performance of OLED, this application discusses the development on preparation of ALD thin film at a low temperature. One concern of ALD is the suppressing effect of ambient temperature on uniformity of thin film. To mitigate this issue, the pumping time in each reaction cycle was increased during the preparation process, which removed reaction byproducts and inhibited the formation of vacancies. As a result, the obtained thin film had both high uniformity and density properties, which provided an excellent encapsulation performance. The results from microstructure morphology analysis, water vapor transmission rate, and lifetime test showed that the difference in uniformity between thin films prepared at low temperatures, with increased pumping time, and high temperatures was small and there was no obvious influence of increased pumping time on light emitting performance. Meanwhile, the permeability for water vapor of the thin film prepared at a low temperature was found to reach as low as 1.5 × 10^−4^ g/(m^2^·day) under ambient conditions of 25 °C and 60% relative humidity, indicating a potential extension in the lifetime for the OLED.

## 1. Introduction

In recent years, research areas and novel applications focusing on organic light emitting devices (OLEDs) have developed rapidly [1,2,3,4]. However, there are still many problems that are inhibiting large scale industrialization of OLEDs. One of the critical bottlenecks is the lifetime of these devices, because of the deleterious impact of water vapor and oxygen on these devices, as first noted by Adachi *et al.* [5]. In the air, the light emitting intensity of OLEDs was found to decrease by 99% within 150 min. When OLEDs worked, it was essential to inject electrons into the cathode, requiring cathode metals with lower work functions such as aluminum, magnesium, calcium. However, these metals readily react with any water vapor which is introduced into the devices, causing the notable decline in OLEDs lifetime. In addition, the water vapor could react with the hole transport layer and electron transport layer, and provide another potential mechanism for device failure [6]. Therefore, effective encapsulation of OLEDs is required to isolate each functional layer from water vapor and oxygen in air, thus prolonging the lifetime of the devices. In recent years, flexible OLEDs have become an area of major research. One of the major issues with flexible OLEDs is that a traditional glass cover plate encapsulation cannot satisfy the flexibility requirement. In lieu of this, thin film encapsulation (TFE) has been introduced to replace traditional glass cover plate encapsulation because dense thin films can be created with sufficient isolation from water and oxygen in the air. TFE can also effectively improve the mechanical properties of the device [7,8].

A number of thin film encapsulation methods have been developed by scholars from different countries. Yamashita *et al.* [9] encapsulated OLEDs via formation of thermal chemical vapor deposition polymer films, which quadrupled the lifetime of the device. Kyokyun [10] took advantage of electron beam physical vapor deposition to prepare magnesium oxide thin films with a thickness of 2100 nm. They also reported an obtained water vapor transmission rate (WVTR) decline to 0.2 g/(m^2^·day), which prolonged the lifetime of prepared OLED device by five times. Huang *et al.* [11] reported that the lifetime of encapsulated OLEDs containing a single-layer of PECVD SiNx was 600 h as compared to 6 h for a bare device. Heeger *et al.* [12] encapsulated devices by spin-coating Cytop^TM^ (a type of perchloro polymer, obtained from Bellex International, Wilmington, DE, USA) as the water vapor barrier, and found that the half-life of the device increased more than five times. Meyer *et al.* [13] prepared a water vapor and oxygen barrier with Al_2_O_3_/ZrO_2_ nano laminates prepared through Atomic Layer Deposition (ALD) method. The technique was found to eliminate pinholes and surface defects significantly in the encapsulated thin film, thus reducing the corrosion of Al_2_O_3_. The thin film deposited via the ALD method was dense and smooth. With a thickness of only 130 nm, they were able to obtain a WVTR of 4.7 × 10^−5^ g/(m^2^·day) at 70% relative humidity at 70 °C [13,14]. Vitex Systems in the United States developed the Barix^TM^ encapsulation technique that alternatively deposited acrylic resin monomer and inorganic thin film to form a flexible OLED which could block permeability of water vapor and oxygen. The performance of Barix^TM^ barrier could be controlled by changing the number of layers and chemical composition of acrylic resin and inorganic material layer. The thin film produced by Barix^TM^ had visible light transmittance higher than 80%, and a roughness average (Ra) of less than 10 nm, with a water vapor transmissivity of 10^−4^–10^−6^ g/(m^2^·day), making Barix^TM^ one of the most recognized encapsulation methods [15]. The reviews on research progress of encapsulation techniques for OLED thin films in recent years show electron beam evaporation, thermal evaporation and sputtering can deposit materials with high melting points, like oxide materials. However, the quality of thin film prepared by these methods is relatively low, and small cracks may occur as the deposited thin film becomes thicker, which can decline gas barrier properties during long-term storage of OLEDs. Compared to these methods, ALD method injects precursors, in the form of pulses, alternately into the reaction chamber. The precursors are uniformly adsorbed on substrate’s surface, and simultaneously form bonds. In one cycle, only one atom layer is formed so that the uniformity of thin film prepared is better and a more dense structure is obtained [16]. The ALD method can accurately control the thickness of the inorganic passivation layer at the nanometer size range, and allows for preparation of a dense and non-porous thin film which isolates the permeability of water vapor and oxygen effectively. Ghosh *et al.* [17] used a single ALD-Al_2_O_3_ layer encapsulation to extend OLED’s lifetime, prepared in over 100 °C. Report from Park *et al.* [18] showed that even with AlO_X_ film of 30 nm would decreased WVTR to 0.0615 g/(m^2^·day). However, the deposition rate of ALD is relatively low, and the sample has to be heated continually for more than 10 h to obtain encapsulation layer with thickness of 100 nm [6]. This raises concerns with OLEDs since organic materials have lower thermal stability than inorganic materials, the long-term heating at the temperature higher than the glass transition temperature of organic material may also cause degradation of devices. Because of this, only the preparation of encapsulation thin film at low temperatures (below the glass transition temperature of organic material) is valuable to the practical application and industrialization of ALD. More reports of ALD thin films are summarized in Table 1, including ALD-Al_2_O_3_ at low temperature. Excellent barrier properties have been report during ALD processes at relatively high temperature. Plasma-enhanced atomic layer deposition, known as PEALD, enable deposition at low temperature, due to its high energetic radicals. However, reports showed that thin films deposited by PEALD exhibit poor WVTR, which was over 10^−2^ g/(m^2^·day). Therefore, improved barrier properties are required with ALD process at low temperature.

**Table 1:** Summary of the barrier properties of thin films including material, deposition condition and barrier performance.

Process	Materials	Deposition condition	Barrier layer structure	WVTR (g/m^2^/day)	OLED lifetime	Ref.
PEALD	Al_2_O_3_:N	TMA, O_2_,N_2_ temp:80 °C	300 nm thick	N/D	650 h 80 °C, 50%	[19]
ALD	Al_2_O_3_	TMA, H_2_O temp:120 °C	25 nm thick	1.7 × 10^−5^ (38 °C)	N/D	[6]
ALD	Al_2_O_3_	TMA, H_2_O temp:80 °C	30 nm thick	0.0615 (90 °C)	193 h	[18]
PEALD	Al_2_O_3_	TMA, O_2_ temp:100 °C	10–40 nm thick	5 × 10^−3^ (RT)	N/D	[20]
PEALD	TiO_2_	TDMAT, O_2_ temp:90 °C	80 nm thick	0.024 (RT)	90 h	[21]

In this article, the thin film that is used as a barrier to water vapor and oxygen was prepared using the ALD method, at low temperatures 80 °C, the surface roughness measured by atomic force microscopy (AFM) deposited at a low temperature were identical to the corresponding values of films deposited by the conventional process at higher temperatures. The ultrathin films showed perfect encapsulation properties, and a low WVTR of 1.5 × 10^−4^ g/(m^2^·day) tested at ambient conditions of 25 °C and 60% relative humidity (RH), could be achieved with thickness of 80 nm, which is truly attractive compared with Barix^TM^ of 5 μm thickness, constructed during complex CVD-UV curing cycles. Compared with Ghosh *et al.* and Park *et al.* [8,17], the film showed relatively better WVTR within process of low temperature conditions. Furthermore, the compatibility of the low temperature Al_2_O_3_ for organic electronics was studied by measuring I-V-L and lifetime of OLEDs. The results demonstrate ALD process at 80 °C, did not cause damage to the organic functional layers, providing a favorable barrier properties for OLEDs. 

## 2. Experimental Section

Growth of Al_2_O_3_ thin films was completed on silicon wafer using the ALD method. The silicon wafer was scrubbed and washed with acetone, ethanol, and deionized water, and then cleaned for 10 min by these solvents under ultrasonication. The silicon wafer was then dried under nitrogen, baked 10 min, and placed into a glove box. The vacuum level in ALD reaction chamber was at the range of 1 × 10^−2^ to 3 × 10^−2^ Pa, and the temperature of the ventilation pipe for ALD equipment was set at 120 °C. The internal temperature of reaction chamber and pumping gas time (PGT) were adjusted during the experiment as needed. Before thin film growth occurs, the substrate, in this instance a silicon wafer, must first been covered with a layer of hydroxyl groups. The precursor, trimethylaluminium (TMA) is injected into the reaction chamber first in the form of pulses, in order to react with hydroxyl groups on surface of silicon wafer. The reaction product (–O–)Al(CH_3_)_2_ is adsorbed on the substrates surface, while gaseous reaction by-products, like CH_4_ and excess TMA precursor, are removed from reaction chamber under the help of inert gas. After the first reaction sequence is completed, water vapor as oxidant, is injected into reaction chamber to react with the adsorbed reaction product in the first reaction sequence. When the reaction reaches saturation, the products and excess moisture are removed from the reaction chamber by inert gas. This allows the monolayer of Al_2_O_3_ with hydroxyl-covered surface to be obtained. The reactions just discussed are shown in the following Formulae (1):

–OH (ad) +Al(CH_3_)_3_ (g) → –O–Al (CH_3_)_2_ (ad) + CH_4_ (g) (1a)

(–O–)Al(CH_3_)_2_ (ad) + H_2_O (g) → (–O–)Al(OH)_2_ (ad) + 2CH_4_ (g) (1b)

In the above formulas, (ad) denotes surface-adsorbed state, and (g) denotes a gaseous state. By repeating above steps, certain thicknesses can be obtained. Since the growth thickness is constant in every cycle, the grown thickness of thin film can be manipulated by controlling the number of cycles completed. In this study, the Al_2_O_3_ thin film was deposited with 2500 cycles at 80 °C, and the multi-point thin film thickness was determined, by ellipsometry, as 223.76 nm (using data fitting, the mean square error (MSE) was 1.501), which was consistent with the prediction by ALD reaction cycles (the predicted thickness of the Al_2_O_3_ thin film after 2500 cycles was 225 nm). 

To determine the ALD reaction window temperature, the measure of decreasing the temperature was adopted together with regulation of PGT to a suitable value. It was found that continual uniform deposition through low temperature ALD could be achieved by extending input time of inert gas and increasing PGT at the low temperature to prevent the pollution between two gas precursors and inhibit any CVD reactions which may occur. For exploring the differences of Al_2_O_3_ thin film performance brought by high and low temperature ALD deposition, Al_2_O_3_ thin films of 50 nm were prepared respectively at 200 °C, PGT = 10 s and 80 °C, PGT = 30 s. The contact angle measurement, SEM observations as well as AFM analysis were carried out to evaluate these differences. The thickness of the deposited Al_2_O_3_ was determined by Woollam variable-angle spectroscopic ellipsometer. Scanning electron microscopy (SEM) was conducted with a field-emission SEM (JSM-6700F, JEOL, Tokyo, Japan) operated at an accelerating voltage of 10 kV, and the samples were coated with a thin layer of gold (5 nm) before analysis. The root-mean-square (RMS) roughness and other surface features of the films were measured with a Veeco AFM (Plainview, NY, USA). Water contact angles were measured using a Kruss contact angle goniometer (Model DSA30), where the sessile drop of 2–3 μL in volume was dispensed with a microsyringe.

The WVTR was measured via electrical analysis on corrosion calcium (Ca) thin film [22]. Through measuring the I–V curves of calcium thin film with different exposure durations in air after encapsulation, the electric resistance can be obtained using equation (2):

(2) $$WVTR\left[g/m^{2}∙\mathrm{day}\right]=-n\times \delta_{\text{Ca}}\times \rho_{\text{Ca}}\times \frac{d}{dt}(\frac{1}{R})\times \frac{\text{M}({\text{H}}_{2}\text{O})}{\text{M}(\text{Ca})}\times \frac{\text{Ca}\_\text{Area}}{\text{Window}\_\text{Area}}$$

where n is the molar ratio (*n* = 2 for this system); δ_Ca_ is the electrical resistivity (3.91 × 10^−8^ Ωm); ρ_Ca_ is the density of calcium (1.55 g/cm^3^); 1/R is the measured electrical conductance; M(H_2_O) and M(Ca) are the molecular weights of water and calcium respectively (18 g/mol and 40 g/mol).

**Figure 1:**
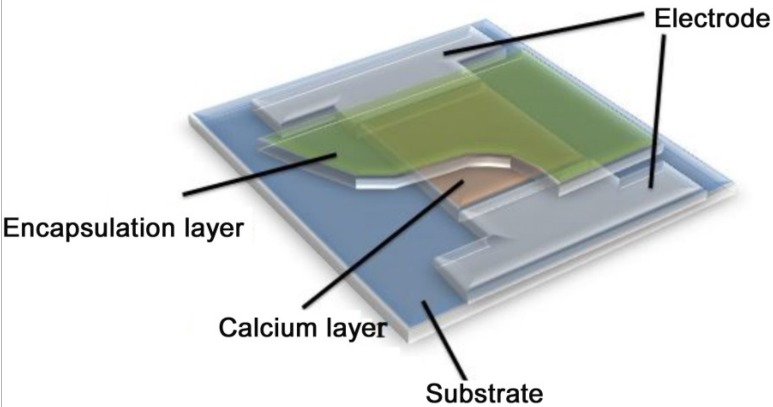
The structure of the device used for the calcium test.

The Ca electrical measuring system was composed of the stable electricity cell controlled by a computer and the probe. The structure of device for calcium test was shown in Figure 1. The 200 nm Ca thin film and 100 nm aluminum electrode were coated onto the glass substrate. The imaged electrode and Ca thin film in this experiment were obtained via addition of a shield template before evaporation. The area of Ca thin film (10 mm × 10 mm) was encapsulated and Al was exposed to act as electrode.

## 3. Results and Discussion

Using the ALD method, 80 nm Al_2_O_3_ thin films were formed on Si substrate at both 80 °C and 200 °C. The water contact angles of both samples were measured and the corresponding surface energies were calculated from results, as shown in Table 2.

**Table 2:** The water contact angles and surface energies of thin films deposited through Atomic Layer Deposition (ALD) under different conditions.

Conditions	Water contact angles	Surface energy (mN/m)
Si(80 °C, PGT = 30 s)	73.4°	39.6 ± 0.2
Si(200 °C, PGT = 10 s)	68.9°	39.8 ± 0.2

The contact angle corresponds, quantitatively, to surface wettability, and the surface energy can be calculated from contact angle using the Owens’ equations [23]. The measurement of the contact angle can indicate the effect of different growth conditions on the formation of thin films since the surface energy is related to surface atom arrangement and internal structures. The data in Table 1 shows that both contact angles of ALD thin film at two temperatures were less than 90°, and that the difference in surface energy were 39.6 ± 0.2 mN/m (80 °C, PGT = 30 s) and 39.8 ± 0.2 mN/m (200 °C, PGT = 10 s), respectively, which verifies that the surface atom arrangement of Al_2_O_3_ thin film deposited via ALD at 80 °C is nearly identical to that of the Al_2_O_3_ thin film deposited via ALD at 200 °C. 

**Figure 2:**
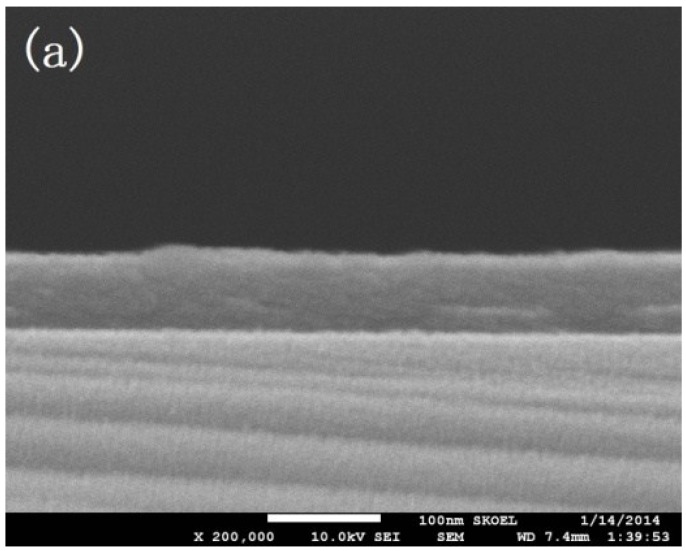

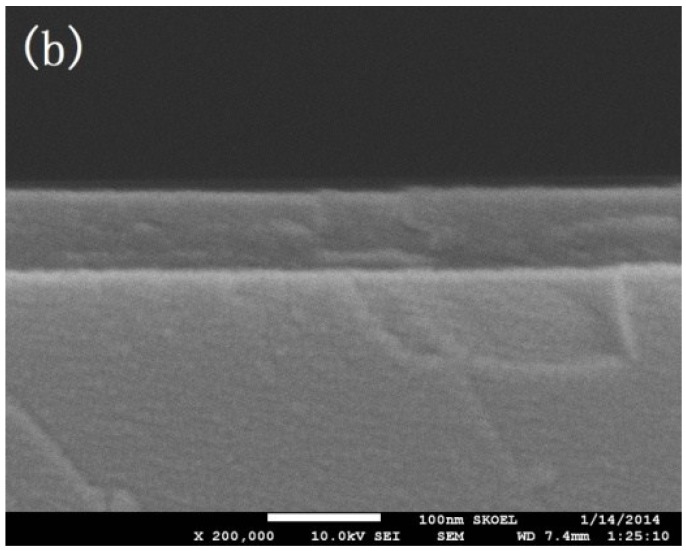
Cross-section SEM images of Al_2_O_3_: (**a**) 80 °C-based Al_2_O_3_; (**b**) 200 °C-based Al_2_O_3_.

**Figure 3:**
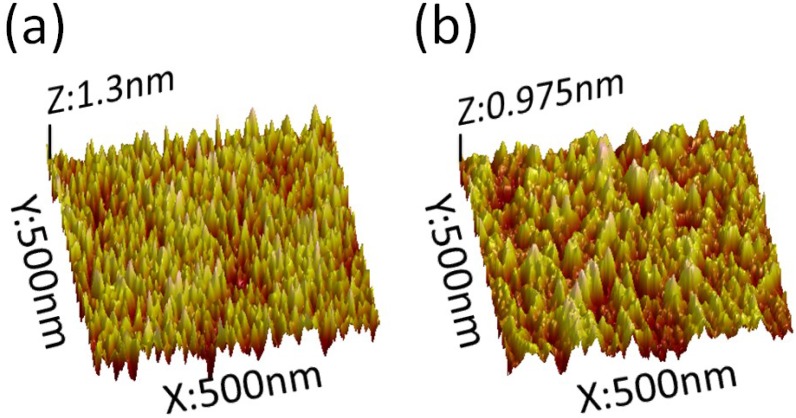
3D Atomic Force Microscopy (AFM) images of Al_2_O_3_ film at deposition conditions: (**a**) 80 °C, PGT = 30 s; and (**b**) 200 °C, PGT = 10 s. The scan area in AFM experiment was 1.0 μm × 1.0 μm.

Figure 2a,b showed the SEM images of cross area of Al_2_O_3_ thin film deposited via low temperature ALD (80 °C, PGT = 30 s) and high temperature ALD (200 °C, PGT = 10 s) respectively, with magnification of 20,000×. Both surfaces of the two thin films were smooth, and the quality of the formed thin films was high. It was also found that the uniformity of the films were close to one another, and neither appeared “S” warped, creased, or cracked. Figure 3a,b shows the AFM morphology of the Al_2_O_3_ thin films deposited via low temperature ALD (80 °C, PGT = 30 s) and high temperature ALD (200 °C, PGT = 10 s), respectively. The results show the roughness of the thin film formed at 80 °C and 200 °C are 0.304 ± 0.030 and 0.278 ± 0.028 nm, respectively. Both of them were found to have similar surface uniformity. This fact implied atomically smooth surface possessed by a stick 2D layer-by-layer growth mode at both higher and lower deposition temperature cases, since the extend PGT at the low temperature to prevent the reaction between two gas precursors and inhibit any CVD reactions which may lead to rough surface [23]. 

**Figure 4:**
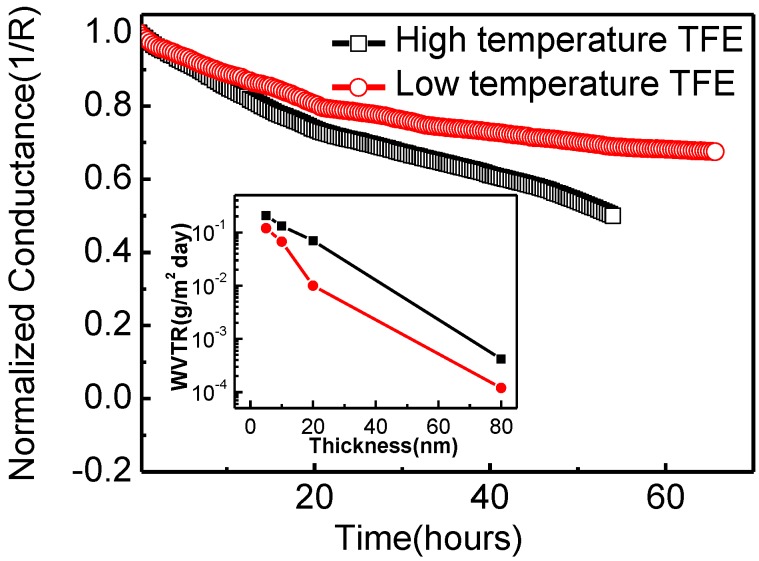
Normalized change in electrical conductance of Ca corrosion tests with (80 °C, PGT = 30 s) Al_2_O_3_ and (80 °C, PGT = 30 s) Al_2_O_3_ as a function of time at 25 °C and 60% RH; the inset showed Water Vapor Transmission Rate (WVTR) changes with different film thicknesses.

The water vapor permeability of Al_2_O_3_ thin films deposited via low temperature ALD (80 °C, PGT = 30 s) and high temperature ALD (200 °C, PGT = 10 s) were measured by the Ca electrical method discussed earlier, and summarized in Figure 4 which depicts the 1/R curve with respect to time in the Ca test, with the black and red lines highlighting the normalized Ca test curves of 80 nm Al_2_O_3_ encapsulation prepared at 80 °C and 80 nm Al_2_O_3_ encapsulation prepared at 200 °C, respectively. The water permeability of the thin films was obtained by calculating the slope of the curve at each time point. Based on the principle of the Ca test, after reaction with water and oxygen, the reacted portion of the Ca electrode has a lower electrical conductance, which will increase the electrical resistance of thin film, as represented by the decrease of conductance in the curve. The calculated WVTRs of single-layered 80 nm-thickness Al_2_O_3_ thin films were 8.6 × 10^−4^ g/(m^2^·day) (200 °C, PGT = 10 s) and 1.5 × 10^−4^ g/(m^2^·day) (80 °C, PGT = 30 s), respectively. The inset of Figure 4 shows WVTRs of both Al_2_O_3_ thin films prepared at 80 °C and 200 °C with different thickness. The comparison between water permeability of encapsulation thin film at low and high temperatures indicated that the thin films prepared at low temperature have higher barrier performance. This result implies that 80 nm-thick 80 °C-PGT 30 s-based Al_2_O_3_ films may have excellent protection properties when integrated on OLEDs.

To further explore the influence of encapsulation thin films on organic devices; OLEDs with a composition of ITO glass/5 nm-thick MoO_3_ layer; 30 nm-thick 4,4′,4″-tris (N-3(3-methylphenyl)-N-phenylamino) triphenyl amine (m-MTDATA) as a hole injection layer; a 20 nm-thick N,N′-biphenyl-N,N′-bis(1-naphenyl)-[1,1′-biphenyl]-4,4′-diamine (NPB) as a hole transport layer; a 50 nm-thick tris-(8-hydroxyquinoline) aluminum (Alq_3_) as a light-emitting layer and an electron transport layer; and a 1 nm-thick LiF capping with 120 nm-thick Al cathode was prepared and encapsulated via 80 nm-thickness low temperature ALD (80 °C; PGT = 30 s). The encapsulating-layer was prepared during the same process with the 80 nm Al_2_O_3_ thin films on Si substrate at 80 °C; ensuring the similarity of properties. The lives of the encapsulated and unencapsulated OLEDs were compared; and the duration decaying from original luminance (1000 cd/m^2^) to 50% was considered as the lifespan of OLED.

**Figure 5:**
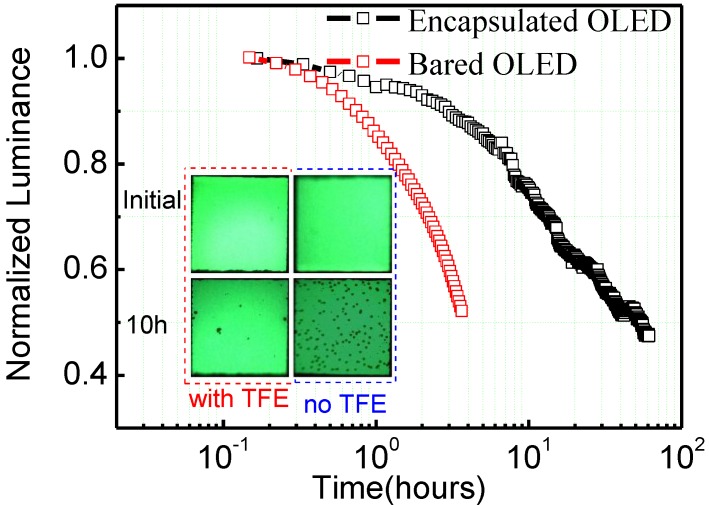
Luminance of OLED encapsulated with and without Al_2_O_3_ thin films as a function of time measured under conditions of 25 °C and 60% RH, the inset shows the photos of the test OLEDs after 10 h.

**Figure 6:**
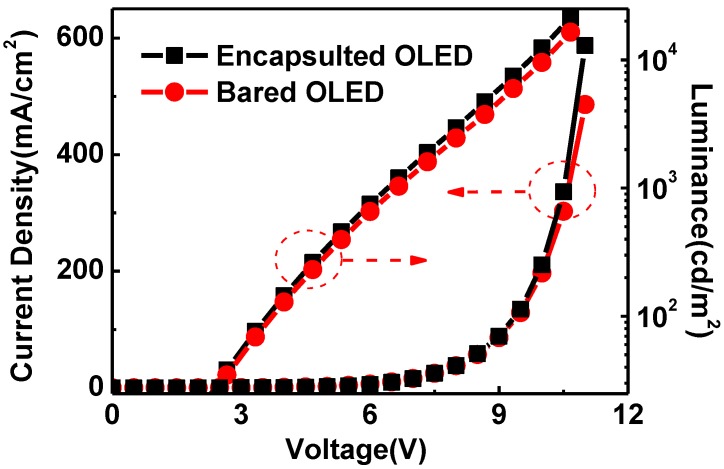
The comparison of I-V characteristics between encapsulated organic light emitting devices (OLED) and bear one.

Figure 5 depicts the life decay curve of the encapsulated and not encapsulated OLEDs. It shows that the encapsulated OLED has a much longer lifespan, over ten times that of the OLED that was not encapsulated. After ten operating hours the encapsulated OLED showed no apparent change in luminance, though there was an obvious decrease for OLED that was not encapsulated. In addition, after examination of the microscopic pictures of the OLED (the inset of Figure 5), better resistance to corrosion from water vapor and oxygen, and less dark stains occurred at light-emitting areas of the encapsulated OLED. Through comparison of I-V curves of the encapsulated and unencapsulated OLED, the influence of low temperature ALD on device was relatively small. In addition, some organics in the OLED crystallized due to the annealing effect at the preparation temperature, which was suitable for transportation of electrical carrier so that the current and luminance of OLED both increased, as shown in Figure 6. However, we must point that the Al_2_O_3_ thin film can react slightly with water vapor to form Al(OH)_3_ [14,24], even this reaction was negligible, the encapsulation properties of Al_2_O_3_ thin film may be compromised during long time operation (several years), potentially leading to the formation of defects and pores, and the initiation of dark stains in light-emitting areas.

## 4. Conclusions

In this article, the ALD technique was applied to prepare encapsulated thin films for OLED. A critical hurdle to overcome was the decreasing of WVTR while preserving the organic materials during the preparation process. By adjusting the temperature of the substrates and PGT, the properties of Al_2_O_3_ thin films through low temperature ALD were found to be similar to those at high temperature. The WVTR of single-layered Al_2_O_3_ thin film at 80 °C was determined by Ca electrical method to be as small as 1.5 × 10^−4^ g/(m^2^·day), which effectively isolated the material from corrosion by water vapor. It was also found that there was no obvious effect of preparation conditions on the performance of these devices. In the next, we attempt to fabricate TFE using various materials, such as Zirconia, Monox,* et al.* This study of PGT represents a critical first step in the realization of low temperature ALD for organic electronics.
